# Fahr’s Disease and its neuropsychiatrist manifestations: A case report

**DOI:** 10.1192/j.eurpsy.2024.1705

**Published:** 2024-08-27

**Authors:** B. Fernández, R. A. Moreira, H. J. Gomes, J. M. Justo

**Affiliations:** Psychiatry, ULSNE, Bragança, Portugal

## Abstract

**Introduction:**

Fahr’s Disease, also known as Fahr’s Syndrome, is a rare genetically dominant disease, characterized by the abnormal accumulation of calcium deposits, or calcifications, in various areas of the brain, particularly the basal ganglia. These calcifications, which are typically bilateral and symmetrical, can lead to a wide range of neurological and psychiatric symptoms, making diagnosis and management challenging. It usually manifests between the ages of 40 and 60, primarily after the age of 30.

**Objectives:**

To contribute to the medical literature by sharing this rare case, thereby increasing awareness and knowledge about Fahr’s Disease among healthcare professionals.

**Methods:**

Non systematic review of the literature and access to the medical history of the patient.

**Results:**

We present a case of a 42 year old woman, who came to our hospital with behavior changes, with increasing confusion and new mystical beliefs, insomnia and agitation.

According to the patient’s husband, the patient sounded confused and inappropriate in her speech. The patient was admitted for evaluation of altered mental status. The patient was alert and oriented to person, place, time, and situation in the emergency department, with shudder while neurologically intact. The patient was unpolite, agitated.

Psychiatry was consulted for evaluation. We decided to admit the patient and did a posterior study with a CT scan and MRI. The MRI, as well as CT scan revealed “dense calcification of the dentate nuclei and the basal ganglia”, highly suggestive of Fahr’s syndrome. The patient’s phosphorus level was 3.5 mg/dl (normal level: 2.5-4.5 mg/dl). Parathyroid hormone (PTH) intact was 53 pg/ml (normal level: 15-65 pg/ml), and calcium level was 10,3 mg/dl (normal level: 8.4-10.5 mg/dl). The vitamin D 25-hydroxy concentration was 43,5 ng/ml (normal level: 30-60 ng/ml).

**Conclusions:**

In conclusion, Fahr’s Disease is a rare and complex neurological disorder characterized by idiopathic calcification of the bilateral basal ganglia, resulting in a diverse range of neurological and psychiatric symptoms. Diagnosis involves clinical evaluation and neuroimaging, while treatment is primarily symptomatic. Further research is needed to better understand the underlying genetic and biochemical mechanisms driving calcification in the brain and to develop more effective therapeutic strategies for this challenging condition.

**Disclosure of Interest:**

None Declared

